# Differential DNA methylation of steatosis and non-alcoholic fatty liver disease in adolescence

**DOI:** 10.1007/s12072-022-10469-7

**Published:** 2023-02-03

**Authors:** Phillip E. Melton, M. A. Burton, K. A. Lillycrop, K. M. Godfrey, S. Rauschert, D. Anderson, G. C. Burdge, T. A. Mori, L. J. Beilin, O. T. Ayonrinde, J. M. Craig, J. K. Olynyk, J. D. Holbrook, C. E. Pennell, W. H. Oddy, E. K. Moses, L. A. Adams, R. C. Huang

**Affiliations:** 1 Menzies Institute for Medical Research, University of Tasmania, 17 Liverpool Street, Private Bag 23, Hobart, TAS 7000 Australia; 2 School of Global and Population Health, The University of Western Australia, Crawley, WA Australia; 3 School of Human Development and Health, Faculty of Medicine, University of Southampton, Southampton, UK; 4 Biological Sciences, Faculty of Natural and Environmental Sciences, University of Southampton, Southampton, UK; 5 NIHR Southampton Biomedical Research Centre, University of Southampton and University Hospital Southampton NHS Foundation Trust, Southampton, UK; 6 MRC Lifecourse Epidemiology Centre, University of Southampton, Southampton, UK; 7 Telethon Kids Institute, The University of Western Australia, Perth, Australia; 8 School of Human Health and Development, Faculty of Medicine, University of Southampton, Southampton, UK; 9 Medical School, The University of Western Australia, Perth, Australia; 10 MCRI, Royal Children’s Hospital, Flemington Road, Parkville, VIC Australia; 11 The Institute for Mental and Physical Health and Clinical Translation (IMPACT), School of Medicine, Deakin University, Geelong, VIC Australia; 12 Department of Gastroenterology and Hepatology, Fiona Stanley and Fremantle Hospitals, Murdoch, WA Australia; 13 School of Medical and Health Sciences, Edith Cowan University, Joondalup, WA Australia; 14 University of Newcastle, Newcastle, NSW Australia; 15 School of Biomedical Sciences, University of Western Australia, Crawley, WA Australia

**Keywords:** Epigenetics, EWAS, *ANK1*, *MIR10A*, *PTPRN2*

## Abstract

**Background and aims:**

Epigenetic modifications are associated with hepatic fat accumulation and non-alcoholic fatty liver disease (NAFLD). However, few epigenetic modifications directly implicated in such processes have been identified during adolescence, a critical developmental window where physiological changes could influence future disease trajectory. To investigate the association between DNA methylation and NAFLD in adolescence, we undertook discovery and validation of novel methylation marks, alongside replication of previously reported marks.

**Approach and results:**

We performed a DNA methylation epigenome-wide association study (EWAS) on DNA from whole blood from 707 Raine Study adolescents phenotyped for steatosis score and NAFLD by ultrasound at age 17. Next, we performed pyrosequencing validation of loci within the most 100 strongly associated differentially methylated CpG sites (dmCpGs) for which ≥ 2 probes per gene remained significant across four statistical models with a nominal *p* value < 0.007. EWAS identified dmCpGs related to three genes (*ANK1, MIR10a*, *PTPRN2*) that met our criteria for pyrosequencing. Of the dmCpGs and surrounding loci that were pyrosequenced (*ANK1 n* = 6, *MIR10a n* = 7, *PTPRN2 n* = 3), three dmCpGs in *ANK1* and two in *MIR10a* were significantly associated with NAFLD in adolescence. After adjustment for waist circumference only dmCpGs in *ANK1* remained significant. These *ANK1* CpGs were also associated with γ-glutamyl transferase and alanine aminotransferase concentrations. Three of twenty-two differentially methylated dmCpGs previously associated with adult NAFLD were associated with NAFLD in adolescence (all adjusted *p* < 2.3 × 10^–3^).

**Conclusions:**

We identified novel DNA methylation loci associated with NAFLD and serum liver biochemistry markers during adolescence, implicating putative dmCpG/gene regulatory pathways and providing insights for future mechanistic studies.

**Graphical abstract:**

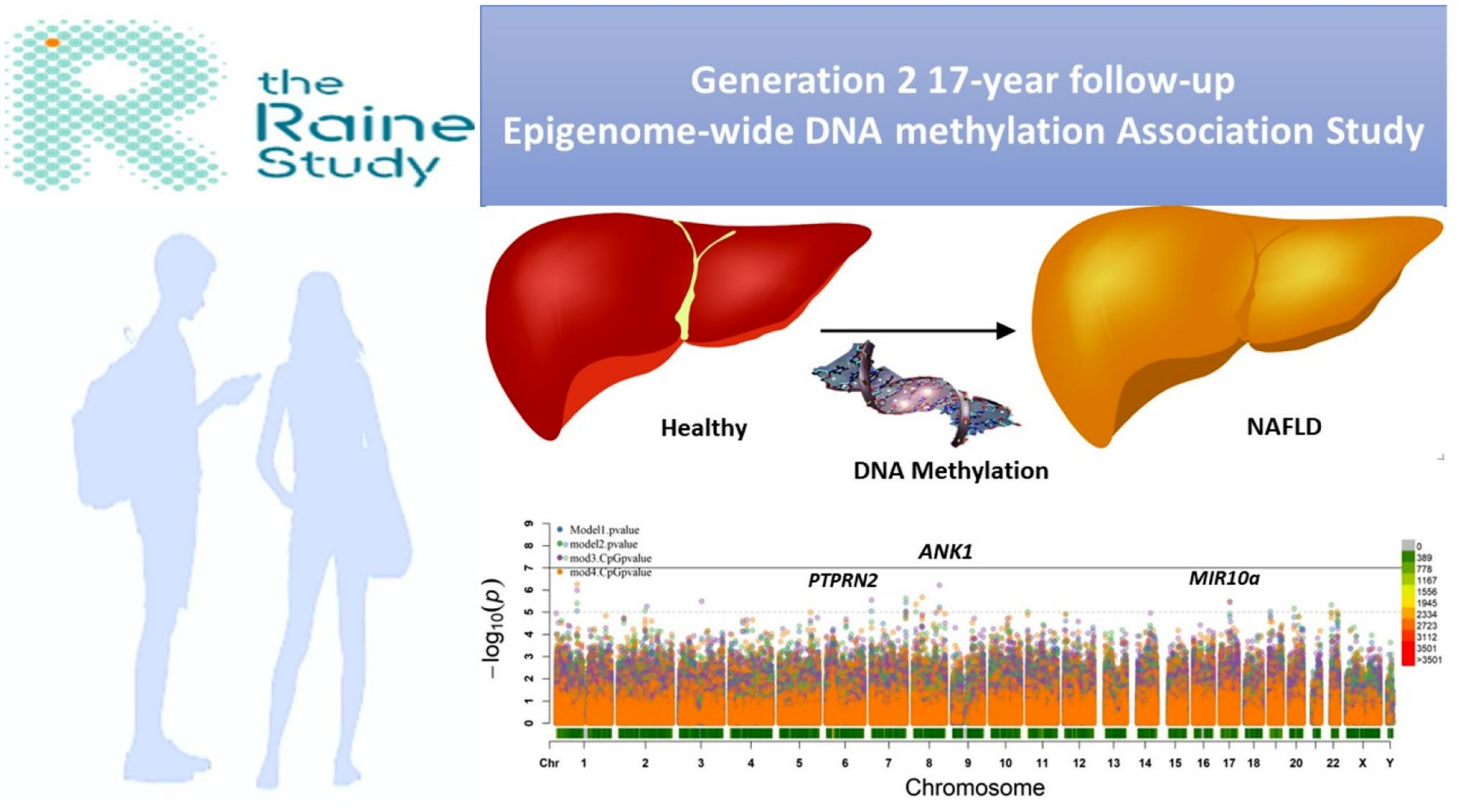

**Supplementary Information:**

The online version contains supplementary material available at 10.1007/s12072-022-10469-7.

## Introduction

Non-alcoholic fatty liver disease (NAFLD) is the most common chronic liver disease in developed countries [1]. NAFLD pathogenesis is thought to be multifactorial, influenced by lifestyle, diet, and genetics [2] but dominated by elevated central adiposity [3].

Epigenetic processes are key components linking environment, genetics, and metabolic disease risk [4]. Previous epigenome-wide DNA methylation association studies (EWAS) have identified differentially methylated CpG sites (dmCpGs) associated with NAFLD in adults [5, 6]. Adolescents and children have been rarely studied [7], but the influences of early life exposures on the epigenome may be clearer and interventions more impactful during times of physiological plasticity [8].

Adolescents with NAFLD may be at the earliest stages of disease and confounders such as alcohol consumption, type 2 diabetes mellitus and other metabolic diseases are less prevalent than in adults. Rapid physiological changes in adolescence denote a period where dysmetabolism may initiate liver damage [9]. Hence, well-characterized adolescent cohorts with a substantial prevalence of NAFLD provide an opportunity to investigate the association between epigenetic variation and NAFLD.

This study aimed to identify dmCpGs in adolescents with NAFLD. We performed a cross-sectional EWAS from whole-blood in the population-based Raine Study where detailed liver assessment had been undertaken at age 17 [10]. The use of whole-blood is well established in EWAS, as it represents a relatively accessible tissue for biochemical analysis in populations studies. Following EWAS, we validated the most strongly associated dmCpGs using pyrosequencing and examined their relationship with additional measures of liver biochemistry (γ-glutamyl transferase (GGT), alanine aminotransferase (ALT), and aspartate aminotransferase (AST)). Finally, we examined DNA methylation of 22 dmCpGs associated with NAFLD in adults [6].

## Methods

### The Raine study

The Raine Study is a longitudinal cohort study initiated 1989–1992 in Perth, Western Australia as a cohort of pregnant women (“*Gen1*”) and their offspring (“*Gen2*”). The Raine Study Gen2 cohort is representative of the general population of Western Australia, as described in detail elsewhere [11]. The current cross-sectional follow-up study was performed when the cohort had reached approximately age 17 years (*Gen2-17*); 1170 participants underwent assessment including (i) a detailed health questionnaire; (ii) anthropometric assessment; (iii) abdominal ultrasonography; and (iv) fasting biochemistry.

### Steatosis score and NAFLD definition

NAFLD was diagnosed by ultrasound-confirmed hepatic steatosis and a daily alcohol consumption < 10 g for females and < 20 g for males [12]. Ultrasound by trained sonographers used a Siemens Antares ultrasound machine with a CH 6–2 curved array probe (Sequoia, Siemens Medical solutions, Mountain View CA), according to a standardized protocol [13]. A single radiologist interpreted images and scored hepatic steatosis severity based upon echotexture, deep attenuation, and vessel blurring (0–1 no steatosis, 2 mild steatosis, and 3–6 moderate-severe steatosis). The intra-observer reliability (κ statistics) for fatty liver was 0.78 (95% confidence interval [CI] 0.73–0.88). Testing for hepatitis B or C virus infections was not performed because notification rates were on average less than 24/100,000 and 23/100,000, respectively, for Western Australian adolescents aged 15–19 years over the study period [12].

### Epigenome-wide DNA methylation profiling

DNA was extracted from blood (Puregene DNA isolation kit; Qiagen, Venlo Netherlands) [14]. Epigenome-wide DNA methylation profiles were undertaken using the Illumina (San Diego, CA) Infinium HumanMethylation 450 BeadChip array (University of British Columbia Centre for Molecular Medicine and Therapeutics; http://www.cmmt.ubc.ca).

Quality control was performed using *shinymethyl* [15], *MethylAID* [16] and *RnBeads* [17] as described previously [18]. Beta-mixture quantile normalization [19] was applied. Technical covariates (plate, slide, well number) were included in all statistical models to adjust for batch effects. Cell counts were estimated using the estimated Houseman method [20] for six cell types (CD8T, CD4T, NK, B cell, monocytes, granulocytes).

### Statistical analysis

#### Univariate analysis

A total of 707 of the original 1,170 Raine Gen2 Age 17 participants who had undergone assessment for NAFLD had complete epigenome and covariate data used for statistical analysis. Univariate comparisons of continuous demographic and biochemical variables with NAFLD status were compared with Student’s *t* or Welch’s one-way tests if normally distributed, and Kruskal–Wallis or Wilcoxon rank sum tests if skewed. Associations of binary variables with NAFLD were assessed using t-tests for parametric variables and Mann–Whitney U tests for non-parametric variables. Measures of adiposity were BMI, and waist circumference, while liver biochemistry comprised serum γ-GGT, ALT, and AST [12]. Insulin-metabolism measures were fasting glucose and insulin, homeostasis model assessment of insulin resistance (HOMA-IR). Serum high-sensitivity C-reactive protein (hsCRP), leptin and adiponectin were measured [12].

#### epigenome-wide DNA methylation association analysis

For EWAS with ultrasound liver steatosis scores, we used linear mixed effects models. Four models were analysed for internal validation: (i) Model 1 adjusted for CpG, age, sex, white blood cell count, principal components derived from genome-wide genotype data, and technical covariates with plate number representing the random effect in the model; (ii) Model 2 included variables from model 1 and Houseman cell count estimates; (iii) Model 3 used all model 2 estimates without principal components; and, (iv) Model 4 included model 1 covariates with assayed white blood counts (red blood cell, neutrophils, lymphocytes, eosinophils, basophils.)

#### Overlap with adult CpGs identified in NAFLD meta-analysis

We investigated 22 dmCpGs previously associated with liver fat accumulation in adults [6]. A Bonferroni correction of *p* value < 0.05/22 = 2.3 × 10^–3^ was used to define statistical significance as we are hypothesis testing if the dmCpGs demonstrate signal at an earlier age.

### Pyrosequencing validation

Inclusion criteria for CpG pyrosequencing were genes represented by 2 or more dmCpGs that were within the top 100 most significantly associated CpGs in statistical model 3 and that were significant across all four statistical models at *p* < 0.007. Four dmCpGs (cg01572694 *MIR10A,* cg05821571 *PTPRN2,* cg19537719 *ANK1,* cg27650870 *ANK1*) in three genes passed these criteria. Sodium bisulphite pyrosequencing was carried out on whole blood DNA samples at age 17 as described [21] (Supplementary Table 1). Pyrosequencing was carried out using PCR products (10 μl) to measure DNA methylation (%) of sixteen dmCpGs (Pyro-Q-CpG 1.0.9 software, Supplementary Table 2) across the three genes of interest (*ANK1, MiR10A, PTPRN2*). Agreement between methylation from pyrosequencing and EWAS arrays was assessed by Bland–Altman plots for four dmCpGs (cg01572694 *MIR10A,* cg05821571 *PTPRN2,* cg19537719 *ANK1,* cg27650870 *ANK1*), one-sample t-test of the difference and linear regression between mean methylation (independent variable) and difference in methylation (dependent variable).

#### Pyrosequencing association analysis

Three statistical models assessed association of DNA methylation of dmCpGs determined by pyrosequencing with steatosis score or NAFLD: (1) model 1 accounted for age and sex; (2) model 2 accounted for age, sex, and five Houseman cell count covariates (CD4T, CD8T, B cell, NK, and monocytes). Granulocytes were removed due to high collinearity with steatosis score and NAFLD [22]; (3) model 3 investigated whether the associated CpG was also influenced by adiposity and included waist circumference as a covariate. These dmCpGs were also investigated if associated with three markers of liver biochemistry (GGT, ALT, AST).

All analyses were performed using the statistical package R, version 3.0 or above.

## Results

### Raine Study Gen2-17: NAFLD phenotype prevalence and characteristics

Table 1 summarizes characteristics of the 707 adolescents who had liver assessments, genome, and epigenome-wide profiling at the Raine Study Gen2-17 follow-up. Overall prevalence of NAFLD was 14.5%, with a higher prevalence in females than males (17.4% vs 11.8%, *p* value = 0.02). NAFLD was associated with higher adiposity, HOMA-IR, serum ALT, GGT and hsCRP, and with lower adiponectin (Table 1).

**Table 1:** Demographic, anthropometric and biochemical phenotypes of NAFLD and non-NAFLD adolescent Raine Study Gen2-17 participants

Characteristic	Non-NAFLD (*n* = 586)	NAFLD (*n* = 121)	*p* value
Female (%)	269 (45.9%)	77 (63.6%)	< 0.001
Age (years)	17.03 (0.26)	17.03 (0.23)	0.992
Adiposity measures (SD)			
Body mass index (kg/m^2^)	21.74 (19.93, 23.74)	28.38 (23.18, 33.01)	< 0.001
Waist circumference (cm)	77.28 (8.57)	91.51 (16.14)	< 0.001
Weight (kg)	65.99 (11)	80.73 (21.38)	< 0.001
Liver function			
ALT (IU/I)	18 (14, 24)	19 (14, 34.5)	0.015
GGT (IU/I)	13 (10, 16)	15 (11, 21.5)	0.001
AST (IU/I)	23 (20, 27)	21.5 (19, 27.75)	0.147
Insulin measures			
Fasting insulin (μU/l)	7.28 (8.85, 10.7)	10.1 (6.57, 17.15)	< 0.001
Fasting glucose (mmol/l)	4.7 (4.5,5)	4.7 (4.5, 5)	0.302
HOMA-IR	1.53 (1.00, 2.21)	2.17 (1.29, 3.38)	< 0.001
hsC-reactive protein (mg/l)	0.49 (0.21, 1.24)	1.35 (0.53, 3.34)	< 0.001
Leptin (ng/ml)	8.1 (2, 22)	24.8 (16.6, 59.10)	< 0.001
Adiponectin (mg/ml)	9.1 (6.48, 12.3)	7.6 (5.2, 10.6)	< 0.001

Parametric variables are presented as mean (standard deviation), nonparametric variables are presented as median (interquartile range) and binomial variables are presented as number (percentage)

### Epigenome-wide DNA methylation association with adolescent NAFLD

**Fig. 1:**
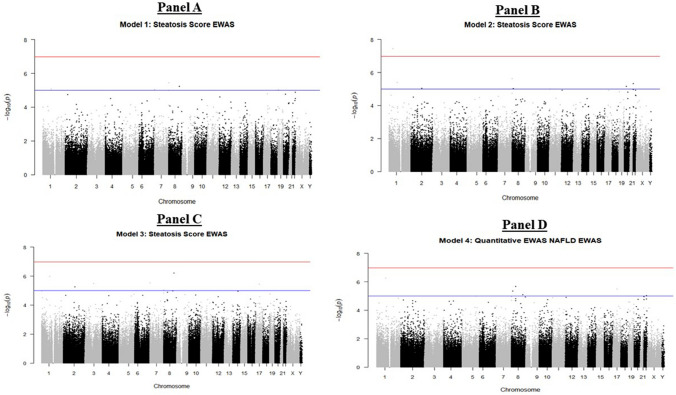
Manhattan plot of − log_10_ *p* value vs. chromosomal position of each dmCpG from the four models used in the epigenome-wide association model. Panel A: Model I was adjusted for dmCpG, age, sex, white cell count, the first two principal components derived from genome-wide genotype data, and technical covariates with the steatosis score as outcome. Panel B: Model II utilized the Houseman count estimates and technical covariates. Panel C: Model III removed the principal components. Panel D: Model IV utilized assayed white blood counts (red blood cell, neutrophils, lymphocytes, eosinophils, basophils) in place of the Houseman Cell Count estimates. Eight CpGs in three genes were identified for follow-up

DNA samples from 707 (52.1% males) adolescents who had undergone ultrasound assessment for NAFLD were analysed for EWAS. Our criteria identified eight dmCpGs in three genes: three dmCpGs (cg19537719, cg27650870, and cg18614735) in ankyrin-1 (*ANK1,* chromosome 8p11), three dmCpGs (cg04514255, cg01572694, and cg15649236) in microRNA 10a (*MIR10A*, chromosome 17q21), and two dmCpGs (cg22676516 and cg05821571) in protein tyrosine phosphatase receptor type N2 (*PTPRN2,* chromosome 7q36) (Fig. 1, Table 2). Supplementary Table 3 shows the fully annotated results for EWAS analysis for all four models.

**Table 2:** Association of pyrosequenced CpG loci from three genes (*ANK1, MIR10A, PTPRN2*) with steatosis score and NAFLD

CpG	Chr	Position	Steatosis score						NAFLD					
			Age/sex			Cell count			Age/sex			Cell Count		
			Beta	95% CI	*p* value	Beta	95% CI	*p* value	OR	95% CI	*p* value	OR	95% CI	*p* value
*ANK1*														
CpG_4 *cg27650870*	8	41583136	0.01	(0.001–0.02)	**0.028**	0.01	(0.001–0.02	**0.008**	1.04	1.01–1.06	**0.003**	1.04	1.01–1.06	**0.001**
CpG_5	8	41583152	0.01	(0.001–0.01)	0.179	0.01	(0.001–0.01)	0.122	1.02	1.00–1.04	0.115	1.02	1.00–1.04	0.083
CpG_6	8	41583161	0.01	(0.001–0.03)	**0.009**	0.02	(0.01–0.03)	**0.003**	1.04	1.01–1.07	**0.004**	1.05	1.02–1.08	**0.002**
CpG_8 *cg19537719*	8	41583498	0.03	(0.02–0.05)	**0.0001**	0.04	(0.02–0.05)	**7.37E**^**−6**^	1.06	1.03–1.10	**0.001**	1.07	1.03–1.11	**0.0003**
CpG_9	8	41583505	0.03	(0.01–0.04)	**5.73E**^**−5**^	0.03	(0.02–0.04)	**5.00E**^**−6**^	1.05	1.02–1.09	**0.001**	1.06	1.03–1.10	**0.0003**
CpG_10	8	41583512	0.03	(0.02–0.04)	**6.10E**^**−5**^	0.03	(0.02–0.04)	**2.30E**^**−6**^	1.06	1.03–1.09	**0.0003**	1.07	1.04–1.10	**2.85E**^**−5**^
*MIR10A*														
CpG_4	17	46657529	− 0.02	(− 0.03)–(− 0.02)	**0.001**	− 0.01	(− 0.02)–(0.01)	0.286	0.96	0.94–0.97	**0.007**	0.98	0.95–1.02	0.349
CpG_5	17	46657532	− 0.04	(− 0.05)–(− 0.02)	**1.90E**^**−5**^	− 0.02	(− 0.04)–0.001	**0.045**	0.93	0.89–0.97	**0.001**	0.96	0.91–1.02	0.185
CpG_6	17	46657535	− 0.02	(− 0.03)–(− 0.02)	**0.001**	− 0.01	(− 0.02)–0.01	0.439	0.96	0.93–0.99	**0.02**	1.00	0.96–1.04	0.926
CpG_7	17	46657538	− 0.04	(− 0.05)–(− 0.02)	**9.70E**^**−6**^	− 0.03	(− 0.05)–(− 0.01)	**0.004**	0.93	0.89–0.96	**0.0001**	0.95	0.90–0.99	**0.03**
CpG_8	17	46657540	− 0.02	(− 0.03)–(− 0.01)	**0.001**	− 0.01	(− 0.02)–0.01	0.308	0.97	0.94–1.00	**0.026**	1.00	0.96–1.03	0.867
CpG_9	17	46657549	− 0.03	(− 0.04)–(− 0.02)	**2.70E**^**−6**^	− 0.02	(− 0.04)–(− 0.01)	**0.009**	0.94	0.91–0.98	**0.001**	0.97	0.93–1.01	0.12
CpG_10 *cg01572694*	17	46657555	− 0.02	(− 0.04)–(− 0.01)	**0.0002**	− 0.01	(− 0.03)–0.0001	0.096	0.95	0.93–0.99	**0.004**	0.97	0.94–1.01	0.178
*PTPRN2*														
CpG_1 *cg05821571*	7	158278927	− 0.03	(− 0.05)–0.0001	**0.044**	− 0.03	(− 0.05)–0.0001	0.05	0.97	0.91–1.03	0.36	0.97	0.91–1.04	0.416
CpG_2	7	158278921	0.01	(− 0.01)–0.04	0.201	0.01	(− 0.01)–0.04	0.229	1.02	0.96–1.08	0.507	1.01	0.96–1.07	0.657
CpG_3	7	158278912	0.01	(− 0.03)–0.05	0.666	0.01	(− 0.03)–0.05	0.784	1.05	0.95–1.16	0.377	1.04	0.93–1.15	0.502

Models were adjusted for age and sex and for estimated cell counts (CD4T, CD8T, B cells, NK cells, monocytes)

*Chr* chromosome, *Position* refers to location of CpG in hg19 build of the human reference genome, *CI* confidence interval, *OR* odds ratio

Values that are statistically significant are highlighted in bold

### Validation by pyrosequencing of NAFLD-associated CpG loci in adolescence

Four dmCpGs were selected for validation by pyrosequencing; cg19537719 and cg27650870 located in the gene body of *ANK1,* cg01572694 located near *MIR10A,* and cg05821571 in the gene body of *PTPRN2.* Supplementary Fig. 1 shows Bland–Altman plots comparing DNA methylation levels measured by the EWAS and pyrosequencing at these CpG loci.

**Fig. 2:**
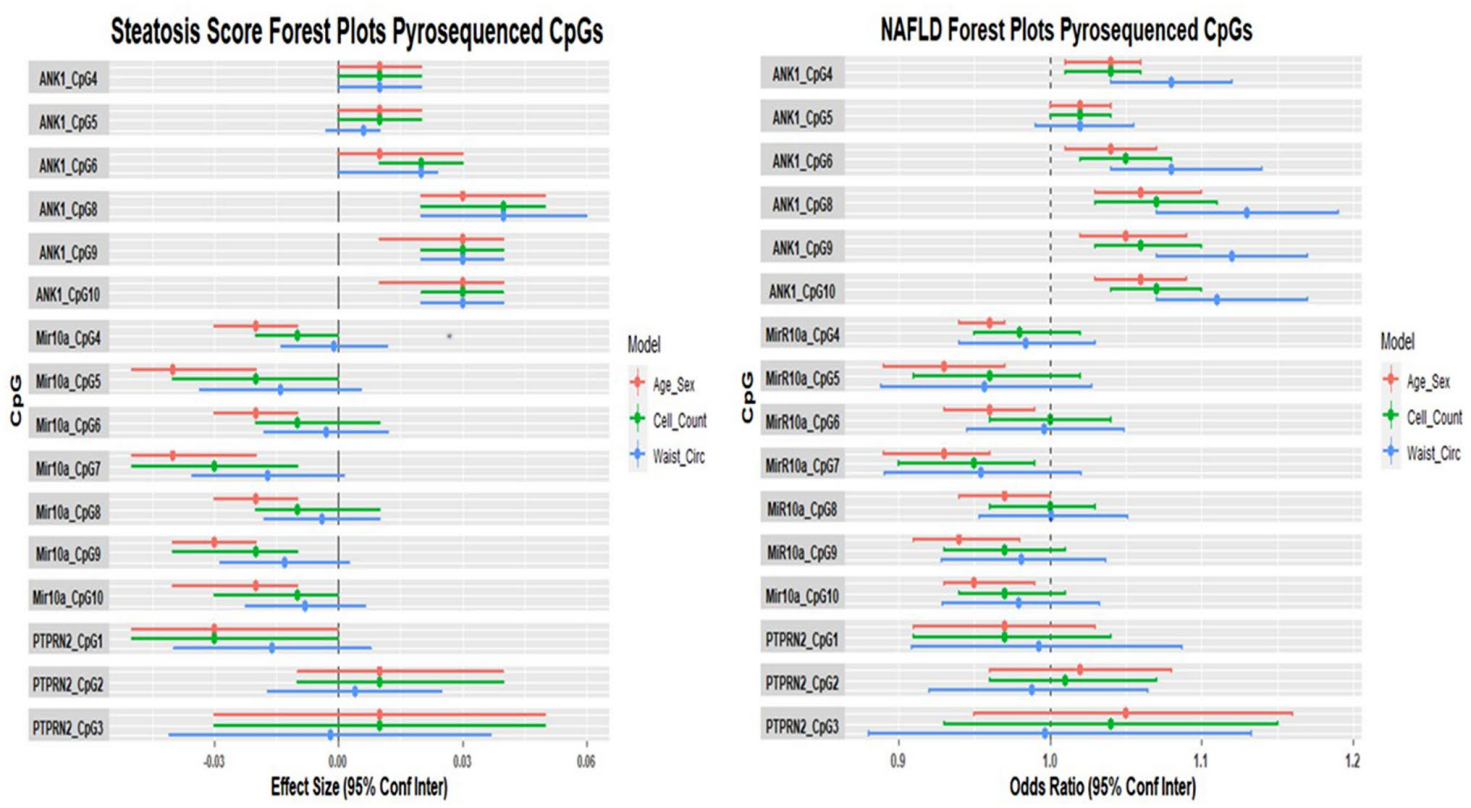
Forest plots of the results from regression models between DNA methylation levels at 16 pyrosequenced CpGs (*ANK1, MIR10A*, *PTPRN2*) on the outcomes of steatosis score and NAFLD. *β*-coefficients for steatosis score, odds ratio for NAFLD, and 95% CI are shown for age and sex (model 1), adjusted for five estimate cell count variables (CD4T, CD8T, B cell, NK cells, monocytes), and accounting for waist circumference

Association results for 16 pyrosequenced dmCpGs from the three genes identified during EWAS are shown in Fig. 2 and Table 2 for steatosis score and NAFLD. Accounting for sex and age, consistent with EWAS results 13 pyrosequenced dmCpGs were associated with steatosis score and 12 dmCpGs with NAFLD. When adjusted for estimated cell type, 8 dmCpGs remained associated with steatosis score and 5 dmCpGs with NAFLD. When waist circumference was included as a measure of adiposity, 6 dmCpGs remained significant for steatosis score and 5 dmCpGs with NAFLD.

*ANK1* CpGs were the most consistent across all outcomes and models, with 5 dmCpGs associated with NAFLD. When waist circumference was included in the model, the *ANK1* CpGs become more significant (Fig. 2). The dmCpG 8:41583512 (*ANK1* CpG_10) was the most significantly associated with NAFLD across all pyrosequencing models.

For *MIR10A*, 3 dmCpGs (cg01572694 (*MIR10*A CpG_10)*, MIR10A* CpG_7, *MIR10A* CpG_9) were associated with steatosis score and one CpG (*MIR10A* CpG_7) associated with NAFLD after cell count adjustment. When waist circumference was included in the steatosis score and NAFLD models none of the *MIR10A* dmCpGs remained significant. None of the three *PTPRN2* dmCpGs that were measured by pyrosequencing were associated with NAFLD. Full results are shown in Table 2.

### Association of CpGs identified by pyrosequencing with additional biochemical markers of liver function

**Fig. 3:**
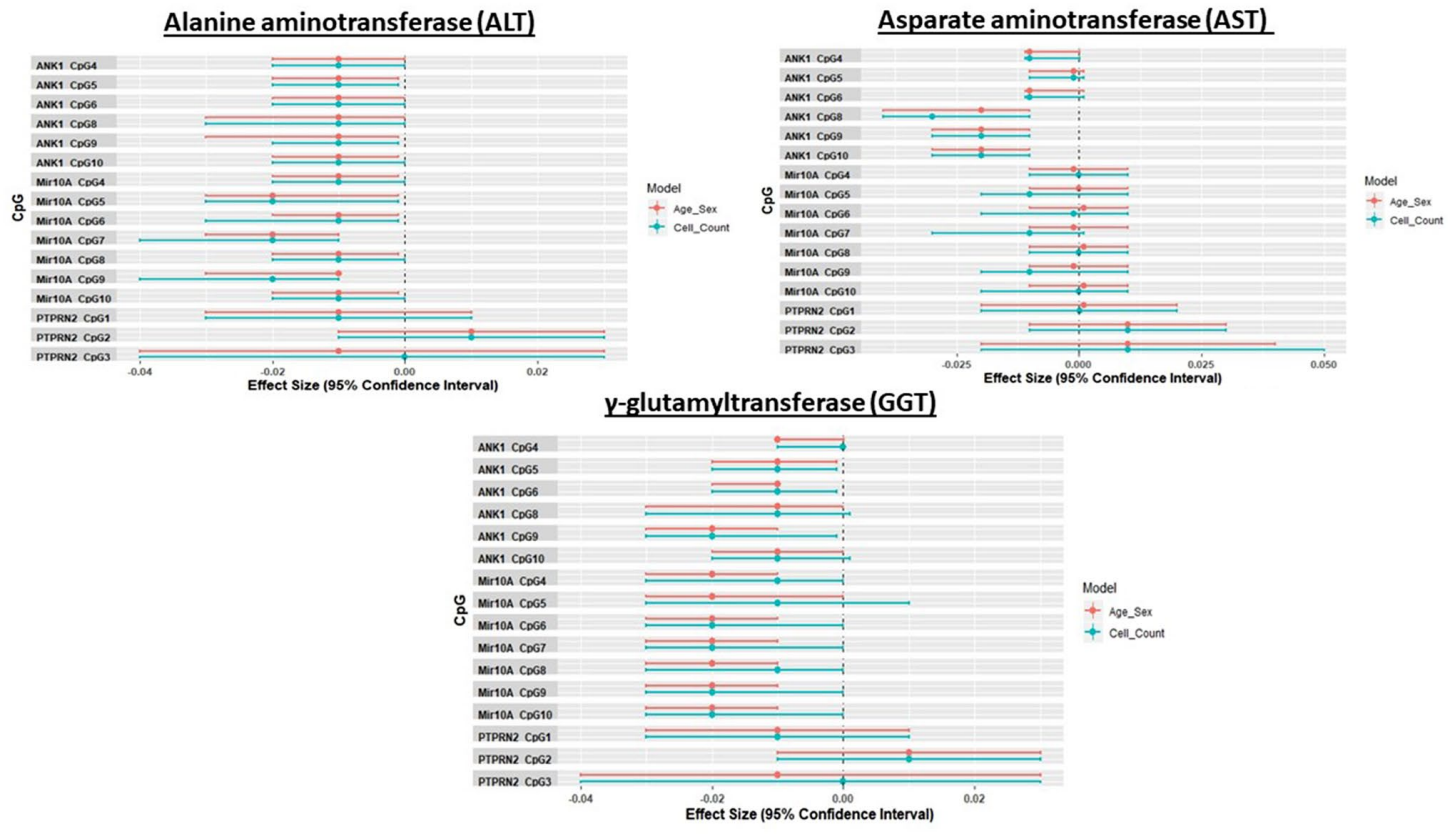
Forest plots of the results from regression models between DNA methylation levels at 16 pyrosequenced CpGs (*ANK1, MIR10A*, *PTPRN2*) on the with liver enzymes (ALT, AST, GGT). β-coefficients and 95% CI are shown for age and sex (model 1) and adjusted for five estimate cell count variables (CD4T, CD8T, B cell, NK cells, monocytes)

We investigated the association of sixteen pyrosequenced CpGs with three liver biochemical markers (GGT, ALT, AST) using linear regression (Supplementary Table 4 and Fig. 3). All seven *MIR10A* CpGs were associated with ALT and GGT when accounting for age and sex. When further adjusted for cell count, four *MIR10A* CpGs demonstrated associations with ALT and six CpGs with GGT. Three *ANK1* CpGs were significant for ALT and AST in both statistical models. For GGT five *ANK1* CpGs were significant for age and sex and three remained associated after cell count adjustment.

### Overlap with adult CpGs identified in adult NAFLD meta-analysis EWAS

We investigated if 22 dmCpGs associated with liver fat in adults [7] were associated with NAFLD in adolescence (Table 3). After correction for multiple testing (Bonferroni correction *p* value < 0.05/2 = 2.3 × 10^–3^), we identified one adult dmCpG (cg11024682) for steatosis score and three dmCpGs (cg14476101, cg26894079, cg11024682) for NAFLD. The dmCpG, cg11024682, were associated with both steatosis score and NAFLD. In addition, dmCpGs cg14476101 and cg26894079 were associated with NAFLD and nominal significance (*p* < 0.05) with steatosis score.

**Table 3:** Association of 22 adult non-alcoholic fatty liver disease associated dmCpGs with steatosis score and NAFLD in adolescence

CpG	Chr	Base pair	Gene	Steatosis score			NAFLD		
				Estimate	SE	*p* value	Estimate	SE	*p* value
cg09469355	1	2161886	*SKI*		1.27	**0.041**	− 0.72	0.4	0.07
cg17901584	1	55353706	*DHCR24*	− 2.04	1.18	0.08	− 0.66	0.37	0.08
cg03725309	1	109757585	*SARS*	− 2.34	1.49	0.12	− 0.87	0.47	0.06
cg14476101	1	120255992	*PHGDH*	− 1.51	0.7	**0.032**	− 0.57	− 2.6	**0.0095**
cg19693031	1	145441552	*TXNIP*	− 0.98	0.97	0.32	− 0.22	0.31	0.47
cg06690548	4	139162808	*SLC7A11*	− 3.22	2.35	0.17	− 0.77	0.74	0.3
cg05119988	4	166251189	*SC4MOL*	0.08	1.08	0.94	− 0.12	0.34	0.72
cg03957124	6	37016869	*COX6A1P2*^a^	− 1.67	1.38	0.23	− 0.86	0.43	**0.04**
cg18120259	6	43894639	*LOC100132354*^a^	− 0.51	1.3	0.7	− 0.41	0.41	0.32
cg17501210	6	166970252	*RPS6KA2*	− 0.27	0.99	0.79	− 0.14	0.31	0.66
cg21429551	7	30635762	*GARS*	− 0.64	0.73	0.38	− 0.13	0.23	0.6
cg11376147	11	57261198	*SLC43A1*	− 0.73	2.51	0.77	− 0.58	0.79	0.46
cg00574958	11	68607622	*CPT1A*	− 3.66	2.58	0.16	− 1.29	0.812	0.11
cg26894079	11	122954435	*ASAM*	− 2.42	1.21	**0.046**	− 0.85	0.381	**0.02**
cg11024682	17	17730094	*SREBF1*	4.11	1.53	**0.0075**	1.13	0.483	**0.01**
cg14020176	17	72764985	*SLC9A3R1*	− 0.03	1.9	0.99	0.16	0.6	0.78
cg19016694	17	80821826	*TBCD*	− 2.8	1.53	0.07	− 0.91	0.48	0.06
cg15860624	19	3811194	*ZFR2*	1.42	0.84	0.09	0.35	0.26	0.19
cg02711608	19	47287964	*SLC1A5*	− 1.23	1.72	0.47	− 0.64	0.54	0.23
cg08309687	21	35320596	*LINC00649*^a^	− 0.95	1.01	0.35	− 0.44	0.32	0.17
cg27243685	21	43642366	*ABCG1*	2.26	1.6	0.16	0.75	0.5	0.14
cg06500161	21	43656587	*ABCG1*	1.24	1.23	0.32	0.28	0.39	0.47

Associations are adjusted for adolescent age, sex, and estimated cell type proportions

*Chr* chromosome, *SE* standard error

^a^Gene names from UCSC Genome Browser build hg19 Additional names taken from paper adult NAFLD EWAS

Nominal (*p* value < 0.05) and Bonferroni corrected (*p* value < 0.023) are shown in bold

## Discussion

We conducted EWAS in a well-characterized cohort of adolescents, to identify specific DNA methylation signatures in whole blood associated with ultrasound-defined NAFLD. We identified dmCpGs in three genes (*ANK1, MIR10A, PTPRN2)* that were associated with steatosis score. Using pyrosequencing, associations in one of these genes (*ANK1*) were confirmed with both steatosis score and NAFLD after accounting for waist circumference and cell count heterogeneity. Further investigation of these specific CpGs with three liver biochemical markers identified CpGs in both *ANK1* and *MIR10A* that were also associated with ALT, AST and GGT, after cell count adjustment. This consistency in the direction of effect for these associations, strongly supports our findings as biological associations.

*ANK1* contains an ankyrin repeat domain, which modulates interactions between cytoskeletal and membrane proteins [23]. ANK1 protein is found in circulating extra-cellular vesicles in animal models of non-alcoholic steatohepatitis (NASH), suggesting a role in cell-to-cell signaling [24]. Genetic variants in *ANK1* are associated with susceptibility to diabetes [25], and in the Mendelian disorder hereditary spherocytosis, a type of hemolytic anemia disease that is known to lead to jaundice, enlarged spleen and liver in pediatric patients [26]. In addition, DNA methylation status of *ANK1* in newborns correlates with maternal pre-pregnancy BMI in humans [27]. We have previously shown that maternal BMI is a significant and independent risk factor for adolescent NAFLD in offspring [9]. Our data suggest that epigenetic changes in *ANK1* may represent a potential link between maternal obesity and subsequent childhood NAFLD; alternatively, this association may be driven by obesity acting on differential DNA methylation and subsequently influencing NAFLD in adolescence [28].

An adult EWAS meta-analysis of NAFLD identified 22 significant dmCpGs [6]. We identified 3 of these 22 dmCpGs as at least nominally significantly associated with NAFLD in adolescents with the same direction of effect. This suggests that adolescence is an important transitional period to better understand the development of NAFLD and may represent the earliest stage of hepatic steatosis or any intermediate stage between childhood and adult NAFLD. While adults and adolescents share risk factors including obesity and insulin resistance, little is known about the role of DNA methylation patterns in liver tissue during childhood or adolescence. Another possibility is the observed DNA methylation is not causing the association but is rather a consequence of NAFLD, as seen in obesity [29]; if so, at the age we examined the duration of exposure to elevated liver fat may not have been sufficient to induce differential DNA methylation for all 22 dmCpGs currently identified in adulthood [7] or other potential differences across the different studies.

A limitation of our study is the modest sample size, limiting statistical power to detect the small effect sizes [30]. However, the Raine Study Gen2-17 is one of the largest population-based cohorts of adolescents with liver ultrasound assessment for NAFLD. Although ultrasound is less sensitive for the detection of minor hepatic steatosis compared with histology, our study utilized a validated and standardized imaging approach for the diagnosis of NAFLD. Our results are supported by overlapping findings with liver enzymes and metabolic risk factors. Furthermore, the European Association for the Study of the Liver recommends liver ultrasound, and not liver biopsy as the preferred initial assessment of individuals suspected of having NAFLD [31]. DNA methylation was measured in whole blood, utilizing estimated cell counts to correct for potential cell type differences, but the DNA methylation of these candidate loci in liver tissue is unknown. However, the overlap of GGT and ALT, well-known surrogates for fatty liver, provides additional confirmation for our findings. We validated our EWAS signal using pyrosequencing in only two (*ANK1, MIR10A)* of the three genes identified; this may be a result of the very high (> 85% methylation) *PPTRN2* gene methylation levels. Lastly, the role of these dmCpGs in regulating transcription is unknown and so inference they are causatively involved in the etiology of NAFLD requires further mechanistic evaluation including the role of DNA methylation in leukocyte differentiation and function.

In summary, we conducted EWAS with hepatic steatosis score and NAFLD in a well characterized adolescent cohort using a two-stage approach. First, we identified dmCpGs relating to three genes that showed differential methylation in adolescents with steatosis score using a genome-wide approach. Second, we validated loci through pyrosequencing and confirmed the associations of loci in one gene with NAFLD (*ANK1*) after accounting for cell count heterogeneity and adiposity. In addition, we investigated the association of these CpGs with traditional liver biochemical markers and found several dmCpGs were associated with GGT and ALT, supporting the previous findings. These findings require replication in additional cohorts and further mechanistic research is needed to identify how changes in *ANK1* methylation influence NAFLD or how NAFLD may influence *ANK1* methylation and gene expression. Based on our informatic analysis, we speculate that methylation changes in CpG sites involved in cell–cell signaling and *MIR10A* controlled TGF-β pathways act during childhood to result in early changes of fatty liver in adolescence, with implications for NAFLD onset and progression in adulthood.

## Supplementary Information

Below is the link to the electronic supplementary material.

Supplementary file1 (DOCX 343 KB)

## Abbreviations

NAFLD: Non-alcoholic fatty liver disease
EWAS: Epigenome DNA methylation study
dmCpG: Differentially methylated CpG site
GGT: γ-Glutamyl transferase
ALT: Alanine aminotransferase
AST: Aspartate aminotransferase
*ANK1*: Ankyrin-1
HOMA-IR: Homeostasis model assessment of insulin resistance
*MIR10a*: MicroRNA 10a
*PTPRN2*: Protein tyrosine phosphatase receptor type N2
hsCRP: High-sensitivity C-reactive protein
BMI: Body mass index
