# Supplementary material for: Differential DNA methylation of steatosis and non-alcoholic fatty liver disease in adolescence
Source: Hepatol Int. 2023 Feb 3;17(3):584–94. doi: 10.1007/s12072-022-10469-7 (PMC9897882; doi:10.1007/s12072-022-10469-7)

**SUPPLEMENTARY MATERIAL**

**Differential DNA methylation and non-alcoholic fatty liver disease in adolescence.**

PE. Melton^1,2,3*^, MA Burton^4^, KA. Lillycrop^5,6^, KM. Godfrey^6,7^, S. Rauschert^8^, D. Anderson^8^, GC. Burdge^9^, TA. Mori^10^, LJ. Beilin^10^, OT. Ayonrinde^10^, JM. Craig^11,12^, JK. Olynyk^13^, JD. Holbrook^7^, CE. Pennell^14^, WH. Oddy^1^, EK. Moses^1,3^, LA. Adams^10^, RC. Huang^8^

^1^Menzies Institute for Medical Research, University of Tasmania, Hobart, Australia.

^2^School of Pharmacy and Biomedical Sciences, Curtin University, Bentley, WA Australia

^3^School of Biomedical Sciences, University of Western Australia, Crawley, WA, Australia

^4^School of Human Development and Health, Faculty of Medicine, University of Southampton, Southampton, U.K.

^5^Biological Sciences, Faculty of Natural and Environmental Sciences, University of Southampton, Southampton, UK.

^6^NIHR Southampton Biomedical Research Centre, University of Southampton and University Hospital Southampton NHS Foundation Trust, Southampton, UK

^7^MRC Lifecourse Epidemiology Unit, University of Southampton, Southampton, UK.

^8^Telethon Kids Institute, University of Western Australia, Perth, Australia.;

^9^Academic Unit of Human Health and Development, University of Southampton;

^10^Medical School, University of Western Australia Perth, Australia.

^11^Early Life Epigenetics Group, MCRI, Royal Children’s Hospital, Flemington Road, Parkville, VIC, Australia

^12^Centre for Molecular and Medical Research, School of Medicine, Deakin University, Geelong, VIC, Australia

^13^Translational Medicine Head of Gastroenterology & Hepatology, Fiona Stanley and Fremantle Hospitals Western Australia.;

^14^University of Newcastle, Newcastle, NSW, Australia

**Supplementary Table 1: PCR Products used for Pyrosequencing of 17 CpGs used in the main text.**

| **Gene and CpG loci** | **Forward primer (5’ - 3’)** | **Reverse primer (3’ - 5’)** | **Sequencing primer (5’ - 3’)** |
| --- | --- | --- | --- |
| **MIR10A CpG 4 -10** | GTGAGAGTTTGTTTTTGGGATTATAAGGT | ACCCTTCCTCCTTTTATACTTAAC | GTAAAGGGAAGAGAATTTTGT |
| **PTPRN2 CpG 7 -5** | GTTTTGTTGGTGGGAGTATGG | AATCACCAACCACCTCCCTTATATAAC | GTGGGAGTATGGTTTGA |
| **ANK1 CpG 8-10**  **ANK1 CpG 4-6** | TTTGGTGGAGGTAGGTTTTTTAG  GTTAGTGGGTTTAGGGGGTAT | CCTAACCAAAAAAAAAAAATACTTTACTCT  AAAACCAAACTATCCAAAAACCCCTCT | GTGTGTTTAGTGTGAAGG  TGTAGGGAGGGTAGTG |

**Supplementary Table 2: Chromosome and position of the 16 CpGs chosen for pyrosequencing.**

| **Gene and CpG loci** | **Chromosomal location (GRCh37/hg19)** |
| --- | --- |
| *MIR10A* CpG 10 **(cg01572694)** | 17:46657555 |
| *MIR10A* CpG 9 | 17:46657549 |
| *MIR10A* CpG 8 | 17:46657540 |
| *MIR10A* CpG 7 | 17:46657538 |
| *MIR10A* CpG 6 | 17:46657535 |
| *MIR10A* CpG 5 | 17:46657532 |
| *MIR10A* CpG 4 | 17:46657529 |
| *PTPRN2* CpG 7 **(cg05821571)** | 7:158278927 |
| *PTPRN2* CpG 6 | 7:158278921 |
| *PTPRN2* CpG 5 | 7:158278912 |
| *ANK1* CpG 10 | 8:41583512 |
| *ANK1* CpG 9 | 8:41583505 |
| *ANK1* CpG 8 **(cg19537719)** | 8:41583498 |
| *ANK1* CpG 6 | 8:41583161 |
| *ANK1* CpG 5 | 8:41583152 |
| *ANK1* CpG 4 **(cg27650870)** | 8:41583136 |

**Supplementary Table 3: Characteristics of CpGs chosen from the epigenome-wide DNA methylation association analysis for validation with pyrosequencing.**

**Supplementary Table 4**: DMRs for NAFLD in relation to adolescent DNA methylation with adjustment for covariates and cell type identified by using both comb-p (p-value<0.01) and DMRcate (FDR<0.01) methods

| **Chromosome: position** | **Gene Name*** | **No. of CpGs in region** | **FDR from DMRcate †** | **P value from comb-p ‡** |
| --- | --- | --- | --- | --- |
| Chr 2: 74875253 - 74875548 | *M1AP* | 8 | 2.25E^-05^ | 2.36E^-11^ |
| Chr 6: 32846947 - 32847811 | *PPP1R2P1* | 24 | 1.57E^-10^ | 2.37E^-11^ |
| Chr 7: 95025611 - 95026538 | *PON3* | 15 | 2.25E^-09^ | 9.98E^-07^ |
| Chr 13: 47471705 - 47472429 | *HTR2a* | 12 | 4.36E^-08^ | 5.96E^-11^ |
| Chr 22: 18267969 - 18268631 |  | 4 | 1.19E^-07^ | 3.78E^-09^ |

∗ DMRcate annotates to UCSC RefGene from Illumina annotation file. The first listed gene is shown.

† DMRcate takes the minimum Benjamini-Hochberg FDR-corrected *P-*value in the region as representative after recalculating *P*-values by using Gaussian kernel smoothing.

‡ Comb-p uses a 1-step Sidak multiple-testing correction on the regional *P* value assigned by using the Stouffer-Liptak method.

**Supplementary Table 5:** Top 10 KEGG pathways enriched for all four statistical models and average of p-values.

|  |  |  | Average | | Model 1 | | Model 2 | | Model 3 | | Model 4 | |
| --- | --- | --- | --- | --- | --- | --- | --- | --- | --- | --- | --- | --- |
| KEGG ID | **Description** | **Size** | **p-value** | **padj** | **p-value** | **padj** | **p-value** | **padj** | **p-value** | **padj** | **p-value** | **padj** |
| 4080 | Neuroactive ligand-receptor interaction | 272 | 0.000974 | **0.042868** | 2.72E-05 | **0.001197** | 0.029398 | 1 | 0.013908 | 0.055633 | 0.211417 | 1 |
| 4514 | Cell adhesion molecules (CAMs) | 133 | 0.105083 | 1 | 0.011956 | 0.131518 | 0.224018 | 1 | 0.13318 | 0.366246 | 0.808169 | 1 |
| 4270 | Vascular smooth muscle contraction | 116 | 0.139873 | 1 | 0.075103 | 0.30041 | 0.157147 | 1 | 0.446651 | 0.890529 | 0.546853 | 1 |
| 4810 | Regulation of actin cytoskeleton | 213 | 0.216342 | 1 | 0.026995 | 0.169683 | 0.603272 | 1 | 0.0038 | 0.02054 | 0.126689 | 1 |
| 5146 | Amoebiasis | 106 | 0.259692 | 1 | 0.058705 | 0.2583 | 0.404482 | 1 | 0.002606 | 0.019875 | 0.426526 | 1 |
| 4145 | Phagosome | 153 | 0.273126 | 1 | 0.034429 | 0.189361 | 0.878482 | 1 | 0.003162 | 0.019875 | 0.397274 | 1 |
| 4670 | Leukocyte transendothelial migration | 116 | 0.308243 | 1 | 0.0086 | 0.126139 | 1 | 1 | 0.00301 | 0.019875 | 0.984434 | 1 |
| 4360 | Axon guidance | 129 | 0.329622 | 1 | 0.168792 | 0.46957 | 0.366369 | 1 | 0.122823 | 0.360279 | 0.305726 | 1 |
| 4020 | Calcium signaling pathway | 177 | 0.47223 | 1 | 0.089439 | 0.302716 | 0.30628 | 1 | 0.182284 | 0.471793 | 0.73487 | 1 |


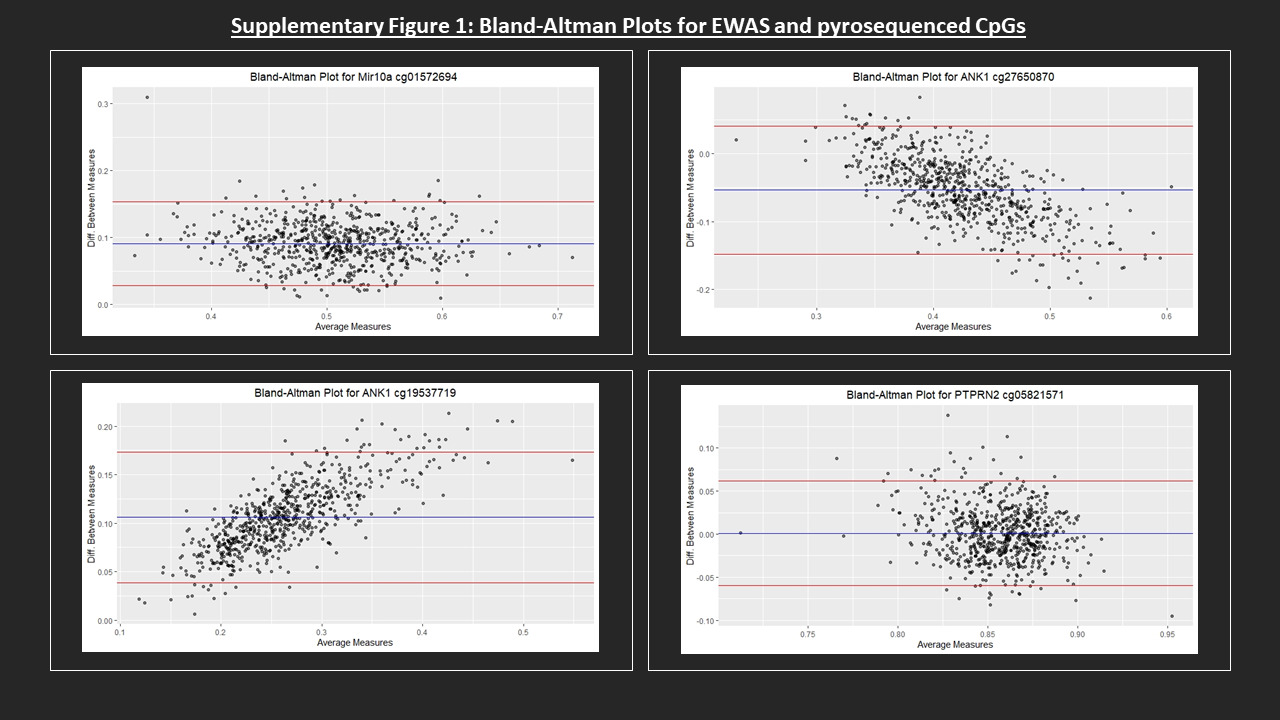

Supplement: Supplementary file 1 — Supplementary file1 (DOCX 343 KB) [file 12072_2022_10469_MOESM1_ESM.docx]
